# Data on selfـــefficacy  and its sources during the COVID-19 crisis:  A Saudi auditor’s perspective

**DOI:** 10.12688/f1000research.146067.1

**Published:** 2024-08-20

**Authors:** Ali Ali Al-Ansi, Saeed Rabea Baatwah, Mahfoudh Hussein Mgammal

**Affiliations:** 1 College of Business Administration, Shaqra University, Afif, Saudi Arabia; 2 College of Administrative Sciences, Seiyun University, Seiyun, Hadhramout, Yemen; 3 Accounting Department, College of Business, Jouf University, Saudi Arabia, Amran University, Amarn, Yemen

**Keywords:** COVID-19; sources of selfـــefficacy; selfـــefficacy; virtual audit; Saudi Arabia.

## Abstract

**Background:**

Auditors during COVID-19 experienced an unprecedented situation where normal audit activities became difficult to conduct. Moreover, COVID-19 forced auditors to introduce a new audit approach, “remote auditing,” which was not common in most audit firms and required the adoption of more advanced technologies. Overall, auditors during COVID-19 needed both cognitive and technical factors to deliver high-quality audits. Despite the challenges, research into how auditors dealt with these issues is limited, presenting an intriguing area of study.

**Methods:**

This dataset provides insights into the Saudi auditor’s experience and beliefs regarding audit activities during COVID-19. Through an online survey, researchers collected data from 193 out of 417 registered auditors with the Saudi Organization for Chartered and Professional Accountants (SOCPA). The survey assessed the auditors’ self-efficacy in conducting audits during the pandemic, exploring its sources and potential moderating factors. Specifically, the dataset includes responses on eight items related to self-efficacy, 19 items covering the four common sources of self-efficacy (mastery experience, vicarious experience, social persuasion, and physiological/emotional states), and six items assessing virtual audit competency. Additionally, the dataset contains demographic information valuable for researchers analysing the relationship between auditor self-efficacy, its sources, and other influencing factors.

**Conclusions:**

Overall, the dataset included in this article may serve a broader audience and be useful in improving several stakeholders’ understanding of the effects of COVID-19 on auditors and how auditors assess their ability to adapt to the COVID-19 consequences. This research contributes to the existing body of knowledge by highlighting the need for auditors to adapt to new circumstances and adopt innovative approaches during challenging times, thereby ensuring the delivery of high-quality audits.

## Introduction

The coronavirus of 2019 (COVID-19) is a health pandemic causing disruption in the lives of humans and corporate businesses. In this crisis, auditors were put in an unprecedented situation to deliver their audit activities, as they used to carry out most audit activities in the present, which became impossible during the pandemic due to social distance and homestay requirements ( Baatwah & Al-Ansi, 2022). Although the COVID-19 crisis raised concerns about the quality of audits and increased the risk of material misstatement ( Kniffin et al., 2020), there has been not much studies addicted to how accounting scholars have collectively contributed to our understanding of the challenges created by the COVID-19 ( Mgammal, Al-Matari, & Bardai, 2022; Rinaldi, 2022). This data article describes external auditors’ status during COVID-19 in Saudi Arabia. In particular, this data article elaborates on auditors’ self-efficacy during COVID-19 and its traditional sources and the degree of proficiency in using virtual audits. Several groups, such as regulators, those using financial data, policymakers, and researchers, may be more interested in this data set.

## Methods

### Ethics and consent statement

The Local Committee of Research Ethics at Shaqra University (HAPO- 01-R-128) has reviewed and evaluated this study on Wednesday 08 May 2024 in accordance with “the rules of research ethics on living organisms” and its executive regulations issued by King Abdulaziz City for Science & Technology (KACST) in 1443 AH.

Due to COVID-19, several individuals and corporate businesses have experienced difficulties conducting their activities as they did prior to the crisis. Auditors are professional audit service providers facing greater challenges in conducting their audit activities ( Mgammal & Al-Matari, 2021; Baatwah & Al-Ansi, 2022; Rinaldi, 2022). These challenges have raised doubts for several stakeholders about the ability of such professional providers to deliver high-quality services ( Arnold, 2020). Thus, this dataset may be used to assess the status and factors that most likely helped the auditors overcome the challenges of COVID-19. In doing so, we surveyed external auditors from Saudi Arabia to assess four constructs related to auditors’ perceptions of the sources of self-efficacy, self-efficacy during COVID-19, and virtual audit proficiency. Specifically, the questionnaire targeted the 417 registered auditors of the 25 audit firms or offices with SOCPA as of April 2021. The questionnaire was shared by email to all registered firms, requesting each firm or office to distribute the questionnaire to all auditors. Between April and August 2021, researchers gathered data from 193 auditors in Saudi Arabia through an online survey, resulting in 193 usable responses.

In designing the questionnaire, we have extensively reviewed related prior research. For example, we used the social cognitive theory developed by Bandura [4] and adapted items from education studies (e.g. Usher & Pajares, 2009; Peura et al., 2021; Zhang, Zhu, & Su, 2023) to develop items related to self-efficacy and its traditional sources because studies on self-efficacy in the context of auditing are limited ( Mohd Sanusi, Iskandar, Monroe, & Saleh, 2018). Furthermore, we referred to the analytical framework of Teeter, Alles, and Vasarhelyi (2010) to create items on virtual audit proficiency. Drawing on existing literature, we identified eight key areas to explore auditor self-efficacy during COVID-19. These areas were measured using eight statements on a five-point Likert scale ranging from “strongly disagree” to “strongly agree.” Similarly, we adapted existing measures for four other factors that influence self-efficacy: mastery experiences (3 items), verbal and social persuasions (4 items), vicarious experiences (3 items), and physiological and emotional states (3 items). These items also used a five-point Likert scale ranging from “strongly disagree” to “strongly agree.” Additionally, we developed six new statements to assess how well auditors can use advanced technologies in their work (virtual audit proficiency). These items were rated on a five-point Likert scale ranging from “rarely” to “always.” The specific wording of all items is provided in Tables 2 and 3. As with any survey study, we included a section providing general information or demographic attributes of the respondents. These include the audit firm’s position, age, education, experiences, the type of affiliated audit firm, and the type of clients. Table 1 reports the demographic attributes of the respondents.

**Table 1.** Features of the population and distribution of frequencies (N=193).

	Frequency	Percentage
**Position within the firm/office**		
Junior auditor	25	13.00%
Auditor	39	20.20%
Senior auditor	30	15.50%
Audit manager	36	18.70%
Partner	63	32.60%
**Gender**		
Male	176	91.20%
Female	17	8.800%
**Age**		
20–29	54	28.00%
30–39	57	29.50%
40–49	40	20.70%
50 and more	42	21.80%
**Education degree**		
Diploma	2	1.000%
Bachelor	124	64.20%
Master	53	27.50%
Ph.D	6	3.100%
Others	8	4.200%
**Major**		
Accounting	188	97.40%
Others	5	2.600%
**Years of experience**		
1-5	76	39.40%
6-10	34	17.60%
11-15	20	10.40%
16 and more	63	32.60%
**Type of audit firm**		
Local	85	44.00%
International	60	31.10%
Big4	48	24.10%
**Type of client**		
Only listed firms	18	9.300%
Only closed firms	58	30.10%
Both types above	67	34.70%
Not involved in either type	50	25.90%
**Participating in auditing during COVID-19**		
Yes	169	87.60%
No	24	12.40%
**In which year-end the audit involvement during COVID-19**		
2019	26	13.50%
2020	14	7.300%
Both years	133	68.90%
Not involved in either year	20	10.40%

Before sharing the questionnaire with the auditors, we have taken several steps to improve our instrument’s readability, validity, and reliability. First, we shared the questionnaire with four audit professors, four audit partners, and two senior auditors to measure the reliability and readability of the content. According to the comments and suggestions, we have amended the questionnaire. Second, given that the first draft of the questionnaire is in English, in consultation with experts, we translated the questionnaire into Arabic, as this is the mother tongue of most auditors in Saudi Arabia. Finally, we filled out all questionnaire items in a Google Form and created a Google Form link to be shared with auditors because social distance restrictions and the preference of individuals communicating remotely were common at the time of developing this questionnaire. All 25 registered audit firms in Saudi Arabia received the online questionnaire via email, using contact information obtained from the SOCPA website. ^1^

### Data description

This article presents data collected from 193 external auditors in Saudi Arabia between April and August 2021. The data, available as raw and filtered versions in a Microsoft Excel worksheet (.xls) and summarized in tables within this article, explores self-efficacy, its sources, and virtual audit competency during the COVID-19 pandemic. The dataset contains the responses of auditors in four major sections. The first section mainly focuses on the demographic characteristics of respondents, asking them to select one option from the multiple-choice answers. This section asked the respondents to indicate, for example, the type of affiliated audit firm, age, experience, type of auditee, and gender. Table 1 reports these features of the population and distribution of frequencies across the respondents.

The second section delves into auditors’ self-efficacy (SE) during COVID-19 through eight statements. Using a five-point Likert scale ranging from “strongly disagree” to “strongly agree,” respondents assessed their confidence in performing their duties during the pandemic. The third section explores the traditional sources that influence self-efficacy. Respondents rated their agreement with three statements related to mastery experience (ME), three statements related to vicarious experience (VE), four statements related to social persuasion (SP), and three statements related to physiological and emotional states (PE), all using the same five-point Likert scale. The final section in this dataset includes six items and focuses on auditors’ competency in using advanced auditing technologies (virtual audit proficiency, or VAP). Using a 5-point scale from “rarely” to “always,” respondents indicated how often they employ advanced technologies for financial statement audit activities in the questionnaire. The items of these constructs and the associated frequency distribution across the Likert-scale are reported in Table 2 and Table 3. We also include the overall response for each construct for each respondent in the dataset. Table 4 reports the descriptive statistics for each construct. The questionnaire was uploaded to the Mendeley Data Repository for more details.

**Table 2.** Constructs’ items for self-efficacy and its sources and frequency distributions for each item as percentage (N=193).

Items No	Acronym	Responses				
		Strongly disagree	Disagree	Not sure	Agree	Strongly agree
**Self-efficacy**						
Q1	SE1	1.55	3.63	13.99	57.51	23.23
Q2	SE2	0.52	5.18	10.88	63.73	19.69
Q3	SE3	1.55	5.18	14.51	49.74	29.02
Q4	SE4	2.07	4.66	16.58	49.22	27.46
Q5	SE5	2.59	6.22	18.65	54.40	18.13
Q6	SE6	2.07	2.59	8.81	59.59	26.94
Q7	SE7	2.07	3.63	9.33	55.44	29.53
Q8	SE8	5.18	22.80	24.35	34.20	13.47
**Mastery-experience**						
Q1	ME1	0.00	5.70	14.51	49.74	30.05
Q2	ME2	0.00	0.52	7.77	58.55	33.16
Q3	ME3	0.00	2.59	15.03	52.85	29.53
**Vicarious-experience**						
Q1	VE1	1.55	4.15	16.06	43.01	35.23
Q2	VE2	0.52	1.55	8.29	54.92	34.72
Q3	VE3	1.04	1.04	5.70	46.11	46.11
**Verbal-persuasion**						
Q1	VP1	2.07	1.55	17.62	48.70	30.05
Q2	VP2	1.55	1.04	21.24	49.74	26.42
Q3	VP3	0.52	1.04	18.65	55.96	23.83
Q4	VP4	0.52	1.04	15.54	55.44	27.46
**Physiological & emotional-states**						
Q1	PE1	16.74	22.80	27.46	25.23	7.77
Q2	PE2	18.13	43.01	21.24	16.06	1.55
Q3	PE3	22.80	32.12	17.10	21.24	6.74

**Table 3.** Items for virtual audit competency and frequency distributions for each item as percentage (N=193).

Items No	Acronym	Response				
		Rarely	Sometimes	Often	Usually	Always
**Virtual audit-proficiency**						
Q1	VAP1	6.7	20.7	19.7	31.1	21.8
Q2	VAP2	8.3	17.1	29.0	29.5	16.1
Q3	VAP3	11.9	26.4	22.8	20.7	18.1
Q4	VAP4	24.9	30.6	20.2	17.6	6.7
Q5	VAP5	15.0	20.2	25.9	27.5	11.4
Q6	VAP6	13.5	12.4	25.4	27.5	21.2

**Table 4.** Descriptive statistics for main constructs.

Construct	Acronym	Mean	Std.	Min	Max
Self-efficacy	SE	3.887	0.730	1.125	5.000
Mastery experience	ME	4.126	0.707	2.000	5.000
Vicarious-experience	VE	4.211	0.696	2.000	5.000
Social-persuasion	SP	4.029	0.736	1.000	5.000
Physiological & emotional-states	PE	2.705	0.940	1.000	5.000
Virtual audit-proficiency	VAP	3.076	0.922	1.000	5.000

### Value of the data

• Capital market authorities, standards-setting authorities, policymakers in firms, and the public can exploit this dataset to assess how external auditors responded to the COVID-19 crisis and how they faced doubts about delivering high-quality audits.
• This dataset will be valuable for researchers interested in studying auditors’ self-efficacy in Saudi Arabia.
• Researchers can use the dataset in this article to provide more insights into how auditors’ demographic attributes can moderate or explain the relationship between self-efficacy and its sources during COVID-19.
• Targeting professional auditors in Saudi Arabia, this dataset provides a unique glimpse into an underexplored environment where socio-economic and cultural factors differ significantly from those prevalent in many other audit markets.
• The dataset can be analyzed and/or compared with data from similar countries as a basis for future comparative studies of auditors’ self-efficacy.

### Ethics and consent statement

The Local Committee of Research Ethics at Shaqra University (HAPO- 01-R-128) has reviewed and evaluated this study in accordance with “the rules of research ethics on living organisms” and its executive regulations issued by King Abdulaziz City for Science & Technology (KACST) in 1443 AH.

Consent: Verbal informed consent was obtained from all participants due to the need for flexibility during the COVID-19 pandemic when face-to-face interactions were limited. The consent process, including the study’s purpose, voluntary participation, and confidentiality measures, was clearly explained to participants. This approach was approved by the Local Committee of Research Ethics at Shaqra University (HAPO-01-R-128) to ensure ethical standards. Participants were informed that they could withdraw from the study at any time without consequences.

## Credit author statement

Baatwah, Saeed was responsible for the research methodology, data curation, and writing of the results and discussion sections. Al-Ansi, Ali contributed to the initial draft and provided review and editing support. Mgammal, Mahfoudh Hussein played a key role in conceptualizing the research, conducting the investigation, and editing the final manuscript.

## Data Availability

Mendeley Data: **Data on self-efficacy and its sources during the COVID-19 crisis: A Saudi auditor’s perspective,** 10.17632/x92c46n2fw.4 The project contains the following underlying data:
• Excel file 1 consists of filtered data for the variables for the questionnaire data sample. Data are available under the terms of the Creative Commons Zero “No rights reserved” data waiver (CC0 1.0 Public domain dedication). Mendeley Data: **Data on self-efficacy and its sources during the COVID-19 crisis: A Saudi auditor’s perspective,** 10.17632/x92c46n2fw.4 The project contains the following underlying data:
• Questionnaire Data are available under the terms of the Creative Commons Zero “No rights reserved” data waiver (CC0 1.0 Public domain dedication).

## Footnotes

^1^
The following Google Form link was shared with the auditors: https://forms.gle/ZB9Pw1GoTJjNY3uo9.
