# Data on selfـــefficacy  and its sources during the COVID-19 crisis:  A Saudi auditor’s perspective

**DOI:** 10.12688/f1000research.146067.2

**Published:** 2024-10-30

**Authors:** Saeed Rabea Baatwah, Ali Ali Al-Ansi, Mahfoudh Hussein Mgammal

**Affiliations:** 1 Accounting Department, College of Business Administration, Dar Al Uloom University, Riyadh, Saudi Arabia; 2 College of Administrative Sciences, Seiyun University, Seiyun, Hadhramout, Yemen; 3 College of Business Administration, Shaqra University, Afif, Saudi Arabia; 4 Accounting Department, College of Business, Jouf University, Saudi Arabia, Amran University, Amarn, Yemen

**Keywords:** COVID-19; sources of selfـــefficacy; selfـــefficacy; virtual audit; Saudi Arabia.

## Abstract

**Background:**

Auditors during COVID-19 experienced an unprecedented situation, in which normal audit activities were difficult to conduct. Moreover, COVID-19 forced auditors to introduce a new audit approach, “remote auditing,” which was not common in most audit firms and required the adoption of more advanced technologies. Overall, auditors during the COVID-19 pandemic needed both cognitive and technical factors to deliver high-quality audits. Despite these challenges, research on how auditors deal with these issues is limited, presenting an intriguing area of study.

**Methods:**

This dataset provides insights into Saudi auditors’ experience and beliefs regarding audit activities during COVID-19. Through an online survey, researchers collected data from 193 of 417 registered auditors with the Saudi Organization for Chartered and Professional Accountants (SOCPA). The survey assessed auditors’ self-efficacy in conducting audits during the pandemic and explored its sources and potential moderating factors. Specifically, the dataset includes responses to eight items related to self-efficacy, 19 items covering four common sources of self-efficacy (mastery experience, vicarious experience, social persuasion, and physiological/emotional states), and six items assessing virtual audit competency. Additionally, the dataset contains demographic information that is valuable for researchers analyzing the relationship between auditor self-efficacy, its sources, and other influencing factors.

**Conclusions:**

Overall, the dataset included in this study may serve a broader audience and be useful in improving several stakeholders’ understanding of the effects of COVID-19 on auditors and how auditors assess their ability to adapt to COVID-19 consequences. This study contributes to the existing body of knowledge by highlighting the need for auditors to adapt to new circumstances and adopt innovative approaches during challenging times, thereby ensuring the delivery of high-quality audits.

## Introduction

The coronavirus disease 2019 (COVID-19) is a health pandemic that disrupts the lives of humans and corporate businesses. Hay, Shires, and Dyk (2021) advocate that the COVID-19 crisis is more likely to affect the auditing profession. In this crisis, auditors were put in an unprecedented situation to deliver their audit activities, as they used to carry out most audit activities in the present, which became impossible during the pandemic due to social distancing and homestay requirements ( Baatwah & Al-Ansi, 2022). Furthermore, the COVID-19 pandemic requires audit firms across the world to revise their assessment of material misstatement risks ( Al-Qadasi, Baatwah, & Omer, 2023). According to the International Auditing and Assurance Standards Board (IAASB) and the Financial Reporting Council (FRC), the COVID-19 pandemic has introduced new risks of material misstatements that require auditors to redesign their audit approaches and execute extensive efforts to reduce these risks ( IAASB, 2020; FRC, 2020). Audit practitioners have also acknowledged several difficulties in conducting audit activities during the COVID-19 pandemic ( PwC, 2020; KPMG, 2020).

Although the COVID-19 crisis raised concerns about the quality of audits and increased the risk of material misstatement, there have not been many studies addressing how accounting scholars have collectively contributed to our understanding of the challenges created by the COVID-19 ( Mgammal, Al-Matari, & Bardai, 2022; Rinaldi, 2022). This article describes the status of external auditors during the COVID-19 pandemic in Saudi Arabia. In particular, this data article elaborates on auditors’ self-efficacy during COVID-19 and its traditional sources, and the degree of proficiency in using virtual audits for Saudi auditors. The accounting and audit profession in Saudi Arabia has recently witnessed considerable development in regulatory frameworks and practices. For example, international accounting standards and international audit standards are references for firms and auditors to practice auditing and accounting activities. It also formulated an independent regulatory body, SCOPA, to develop and monitor auditing accounting practices in Saudi Arabia. However, similar to auditors in other countries, Saudi auditors experienced anomalies in conducting audit activities during the pandemic. Thus, several groups, such as regulators, those using financial data, policymakers, and researchers, may be more interested in this dataset.

## Methods

Due to COVID-19, several individuals and corporate businesses have experienced difficulties in conducting their activities as they did prior to the crisis. Auditors are professional audit service providers that face greater challenges in conducting their audit activities ( Mgammal & Al-Matari, 2021; Baatwah & Al-Ansi, 2022; Rinaldi, 2022). These challenges have raised doubts regarding the ability of professional providers to deliver high-quality services ( Arnold, 2020). Thus, this dataset can be used to assess the status and factors that most likely help auditors overcome the challenges of COVID-19. We surveyed external auditors from Saudi Arabia to assess four constructs related to auditors’ perceptions of the sources of self-efficacy, self-efficacy during COVID-19, and virtual audit proficiency.

We identified all registered Saudi auditors as our population framework. SOCPA, the accounting and audit profession regulatory body in Saudi Arabia, provides a list of registered auditors and their information. As of April 2021, the list of registered auditors includes 25 audit firms/offices associated with 417 auditors, including audit partners and audit managers. Thus, all registered Saudi auditors were our population in this study, and they all had the chance to participate in this study. Specifically, the questionnaire targeted 417 registered auditors of 25 audit firms or offices with SOCPA as of April 2021. The online questionnaire was shared by email with all registered firms requesting each firm or office to distribute the questionnaire to all auditors. To increase the number of participants, we emailed audit firms and offices with two gentle reminders. Between April and August 2021, researchers gathered data from 193 auditors in Saudi Arabia, comprising 46% of the entire population. We assigned two researchers to review all responses for incompleteness and for inappropriateness of answers. This review concluded that 193 responses were usable for analysis. Furthermore, the researchers verified the potential non-response bias issue by comparing the responses received in April with those received in August. Using a two-tailed t-test, the researchers found no variation in the responses across the two groups, suggesting the absence of this issue.

In designing the questionnaire, we extensively reviewed the related prior research. For example, we used the social cognitive theory developed by Bandura [4] and adapted items from education studies (e.g. Usher & Pajares, 2009; Peura et al., 2021; Zhang, Zhu, & Su, 2023) to develop items related to self-efficacy and its traditional sources because studies on self-efficacy in the context of auditing are limited ( Mohd Sanusi, Iskandar, Monroe, & Saleh, 2018). Furthermore, we referred to Teeter et al.’s (2010) analytical framework to create items on virtual audit proficiency. Given that the questionnaire items are used in prior literature from other domains, the adaption of these items involves wording modifications to fit the auditing context. However, the adaptation of these items was shared with professors and experienced audit partners.

Drawing on existing literature, we identified eight key items to explore auditor self-efficacy during COVID-19. These items are measured using eight statements on a five-point Likert scale ranging from “strongly disagree” to “strongly agree.” Similarly, we adapted existing measures for the four factors that influence self-efficacy: mastery experiences (3 items), verbal and social persuasion (4 items), vicarious experiences (3 items), and physiological and emotional states (3 items). These items also used a five-point Likert scale, ranging from “strongly disagree” to “strongly agree.” In addition, we developed six new statements to assess how well auditors can use advanced technologies in their work (virtual audit proficiency). These items were rated on a five-point Likert scale ranging from “rarely” to “always.” The specific wording of all the items is provided in Tables 2 and 3. As in any survey study, we included a section providing general information or demographic attributes of the respondents. These include the audit firm’s position, age, education, experience, type of affiliated audit firm, and client type. Table 1 presents the respondents’ demographic attributes.

**Table 1.** Features of the population and distribution of frequencies (N=193).

	Frequency	Percentage
**Position within the firm/office**		
Junior auditor	25	13.00%
Auditor	39	20.20%
Senior auditor	30	15.50%
Audit manager	36	18.70%
Partner	63	32.60%
**Gender**		
Male	176	91.20%
Female	17	8.800%
**Age**		
20–29	54	28.00%
30–39	57	29.50%
40–49	40	20.70%
50 and more	42	21.80%
**Education degree**		
Diploma	2	1.000%
Bachelor	124	64.20%
Master	53	27.50%
Ph.D	6	3.100%
Others	8	4.200%
**Major**		
Accounting	188	97.40%
Others	5	2.600%
**Years of experience**		
1-5	76	39.40%
6-10	34	17.60%
11-15	20	10.40%
16 and more	63	32.60%
**Type of audit firm**		
Local	85	44.00%
International	60	31.10%
Big4	48	24.10%
**Type of client**		
Only listed firms	18	9.300%
Only closed firms	58	30.10%
Both types above	67	34.70%
Not involved in either type	50	25.90%
**Participating in auditing during COVID-19**		
Yes	169	87.60%
No	24	12.40%
**In which year-end the audit involvement during COVID-19**		
2019	26	13.50%
2020	14	7.300%
Both years	133	68.90%
Not involved in either year	20	10.40%

Before sharing the questionnaire with auditors, we took several steps to improve our instrument’s readability, validity, and reliability. First, the questionnaire was shared with four audit professors, four audit partners, and two senior auditors to measure the reliability and readability of the content. Based on these comments and suggestions, we amended the questionnaire. Second, given that the first draft of the questionnaire was in English, in consultation with experts, we translated the questionnaire into Arabic, as this is the native language of most auditors in Saudi Arabia. Finally, we filled out all questionnaire items in a Google Form and created a Google Form link to be shared with auditors because social distance restrictions and the preferences of individuals communicating remotely were common at the time of developing this questionnaire. Furthermore, using a Google Form link is a common method for collecting data in survey-based studies as it is associated with less effort and fewer errors. Thus, all 25 registered audit firms in Saudi Arabia received an online questionnaire via email using contact information obtained from the SOCPA website. ^1^

### Data description

This study presents data collected from 193 external auditors in Saudi Arabia between April and August 2021. The data were available in raw and filtered versions in a Microsoft Excel worksheet (.xls) and summarized in tables within this article, explores self-efficacy, its sources, and virtual audit competency during the COVID-19 pandemic. The dataset contains auditors’ responses in the four major sections. The first section mainly focused on the demographic characteristics of respondents, asking them to select one option from the multiple-choice answers. This section asked respondents to indicate, for example, the type of affiliated audit firm, age, experience, type of auditee, and gender. Table 1 reports the features of the population and the distribution of frequencies across respondents.

The second section delves into auditors’ self-efficacy (SE) during the COVID-19 pandemic through eight statements. Using a five-point Likert scale ranging from “strongly disagree” to “strongly agree,” respondents assessed their confidence in performing their duties during the pandemic. The third section explores the traditional sources that influence self-efficacy. Respondents rated their agreement with three statements related to mastery experience (ME), three statements related to vicarious experience (VE), four statements related to social persuasion (SP), and three statements related to physiological and emotional states (PE), using the same five-point Likert scale. The final section of this dataset includes six items and focuses on auditors’ competency in using advanced auditing technologies (virtual audit proficiency or VAP). Using a 5-point scale from “rarely” to “always,” respondents indicated how often they employ advanced technologies for financial statement audit activities in the questionnaire. The items of these constructs and the associated frequency distribution across the Likert scale are reported in Tables 2 and 3, respectively. We also included the overall responses for each construct for each respondent in the dataset. Table 4 reports the descriptive statistics for each construct using basic statistics such as the mean, standard deviation, maximum, and minimum. The questionnaire was uploaded to the Mendeley Data Repository for further details.

**Table 2.** Constructs’ items for self-efficacy and its sources and frequency distributions for each item as percentage (N=193).

Items No	Acronym	Responses				
		Strongly disagree	Disagree	Not sure	Agree	Strongly agree
**Self-efficacy**						
Q1	SE1	1.55	3.63	13.99	57.51	23.23
Q2	SE2	0.52	5.18	10.88	63.73	19.69
Q3	SE3	1.55	5.18	14.51	49.74	29.02
Q4	SE4	2.07	4.66	16.58	49.22	27.46
Q5	SE5	2.59	6.22	18.65	54.40	18.13
Q6	SE6	2.07	2.59	8.81	59.59	26.94
Q7	SE7	2.07	3.63	9.33	55.44	29.53
Q8	SE8	5.18	22.80	24.35	34.20	13.47
**Mastery-experience**						
Q1	ME1	0.00	5.70	14.51	49.74	30.05
Q2	ME2	0.00	0.52	7.77	58.55	33.16
Q3	ME3	0.00	2.59	15.03	52.85	29.53
**Vicarious-experience**						
Q1	VE1	1.55	4.15	16.06	43.01	35.23
Q2	VE2	0.52	1.55	8.29	54.92	34.72
Q3	VE3	1.04	1.04	5.70	46.11	46.11
**Verbal-persuasion**						
Q1	VP1	2.07	1.55	17.62	48.70	30.05
Q2	VP2	1.55	1.04	21.24	49.74	26.42
Q3	VP3	0.52	1.04	18.65	55.96	23.83
Q4	VP4	0.52	1.04	15.54	55.44	27.46
**Physiological & emotional-states**						
Q1	PE1	16.74	22.80	27.46	25.23	7.77
Q2	PE2	18.13	43.01	21.24	16.06	1.55
Q3	PE3	22.80	32.12	17.10	21.24	6.74

**Table 3.** Items for virtual audit competency and frequency distributions for each item as percentage (N=193).

Items No	Acronym	Response				
		Rarely	Sometimes	Often	Usually	Always
**Virtual audit-proficiency**						
Q1	VAP1	6.7	20.7	19.7	31.1	21.8
Q2	VAP2	8.3	17.1	29.0	29.5	16.1
Q3	VAP3	11.9	26.4	22.8	20.7	18.1
Q4	VAP4	24.9	30.6	20.2	17.6	6.7
Q5	VAP5	15.0	20.2	25.9	27.5	11.4
Q6	VAP6	13.5	12.4	25.4	27.5	21.2

**Table 4.** Descriptive statistics for main constructs.

Construct	Acronym	Mean	Std.	Min	Max
Self-efficacy	SE	3.887	0.730	1.125	5.000
Mastery experience	ME	4.126	0.707	2.000	5.000
Vicarious-experience	VE	4.211	0.696	2.000	5.000
Social-persuasion	SP	4.029	0.736	1.000	5.000
Physiological & emotional-states	PE	2.705	0.940	1.000	5.000
Virtual audit-proficiency	VAP	3.076	0.922	1.000	5.000

### Value of the data

• Capital market authorities, standards-setting authorities, policymakers in firms, and the public can use this dataset to assess how external auditors respond to the COVID-19 crisis and how they face doubts about delivering high-quality audits.
• This dataset will be valuable for researchers interested in studying auditor self-efficacy in Saudi Arabia.
• Researchers can use the dataset in this study to provide more insights into how auditors’ demographic attributes can moderate or explain the relationship between self-efficacy and its sources during COVID-19.
• Targeting professional auditors in Saudi Arabia, this dataset provides a unique glimpse into an underexplored environment where socioeconomic and cultural factors differ significantly from those prevalent in many other audit markets.
• The dataset can be analyzed and/or compared with data from similar countries as a basis for future comparative studies of auditors’ self-efficacy.

### Ethics and consent statement

The Local Committee of Research Ethics at Shaqra University (HAPO- 01-R-128) reviewed and evaluated this study on Wednesday May 08, 2024, in accordance with “the rules of research ethics on living organisms” and its executive regulations issued by King Abdulaziz City for Science and Technology (KACST) in 1443 AH.

Verbal informed consent was obtained from all participants because of the need for flexibility during the COVID-19 pandemic when face-to-face interactions were limited. The consent process, including the purpose of the study, voluntary participation, and confidentiality measures, was clearly explained to the participants. This study was approved by the Local Committee of Research Ethics at Shaqra University (HAPO-01-R-128) to ensure ethical standards. The participants were informed that they could withdraw from the study at any time without any consequences.

## Credit author statement

Baatwah, Saeed were responsible for the research methodology, data curation, and writing the results and discussion sections. Al-Ansi, Ali contributed to the initial draft, and provided review and editing support. Mgammal, Mahfoudh Hussein played a key role in conceptualizing the research, conducting the investigation, and editing the final manuscript.

## Data Availability

Mendeley Data: **Data on self-efficacy and its sources during the COVID-19 crisis: A Saudi auditor’s perspective,** DOI: 10.17632/x92c46n2fw.4 ( Al-Ansi et al., 2024). The project contains the following underlying data:
• Excel file 1 consists of filtered data for the variables in the questionnaire data sample. • Questionnaire. Mendeley Data: **Data on self-efficacy and its sources during the COVID-19 crisis: A Saudi auditor’s perspective,** DOI: 10.17632/x92c46n2fw.4 ( Al-Ansi et al., 2024). Data are available under the terms of the Creative Commons Zero “No rights reserved” data waiver (CC0 1.0 Public domain dedication).

## Footnotes

^1^
The following Google Form link was shared with the auditors: https://forms.gle/ZB9Pw1GoTJjNY3uo9.
