# Supplementary material for: Data on selfـــefficacy  and its sources during the COVID-19 crisis:  A Saudi auditor’s perspective
Source: F1000Res. 2024 Oct 30;13:935. Originally published 2024 Aug 20. [Version 2] doi: 10.12688/f1000research.146067.2 (PMC11538592; doi:10.12688/f1000research.146067.2)
Supplement: Peer review history

# Reviewer report: Reviewer response for version 2

**DOI:** 10.5256/f1000research.172543.r336506

**Published:** 2024-11-09

**Authors:** Che Siti Lazrina Md Lazim

**Affiliations:** 1 Commerce and Accountancy, Universiti Tunku Abdul Rahman, Kampar, Perak, Malaysia

In summary, the authors addressed all the comments by revising language, adding global context, clarifying the survey development process, and improving the overall presentation of the manuscript.:

 Phrase Revision:

 Issue: The phrase "there has been not much studies addicted to" was awkward and unclear.

 Response: The phrase was revised to "there have not been many studies addressing" to improve clarity and accuracy.

 Context on COVID-19's Global Impact on Auditing:

 Issue: The manuscript should provide a brief overview of the global impact of COVID-19 on auditing before focusing on Saudi Arabia.

 Response: The introduction was expanded to include a global context on the impact of COVID-19 on auditing, highlighting challenges and regulatory concerns worldwide. Additionally, more information was added about the development of the accounting and auditing profession in Saudi Arabia.

 Survey Development Process:

 Issue: The methods section lacked a clear explanation of the survey's development process, including how items were selected and validated.

 Response: The methods section was clarified by providing detailed information on how questionnaire items were adapted from prior research and validated. Specific steps in the process of questionnaire development, item selection, and the population framework were added, along with details on data collection and response verification.

 Language Consistency and Clarity:

 Issue: There were minor language inconsistencies and awkward phrasing. For example, the mention of "The Local Committee of Research Ethics at Shaqra University (HAPO-01-R-128)" was not seamlessly integrated.

 Response: The statement regarding the ethics committee was moved to the "Ethics and Consent Statement" section, and the manuscript was proofread to improve language and overall clarity.

Are sufficient details of methods and materials provided to allow replication by others?

Partly

Is the rationale for creating the dataset(s) clearly described?

Yes

Are the datasets clearly presented in a useable and accessible format?

Yes

Are the protocols appropriate and is the work technically sound?

Partly

Reviewer Expertise:

Accounting and Education Technology , Small Medium Enterprises in Accounting Issue

I confirm that I have read this submission and believe that I have an appropriate level of expertise to confirm that it is of an acceptable scientific standard.

# Reviewer report: Reviewer response for version 2

**DOI:** 10.5256/f1000research.172543.r336507

**Published:** 2024-11-08

**Authors:** Jaizah Othman

**Affiliations:** 1 Imam Abdulrahman Bin Faisal University, Dammam, Eastern Province, Saudi Arabia

The revised article has been strengthened by clarifying language and structure, particularly in the introduction and methods sections, where a global perspective on COVID-19’s impact on auditing was added before focusing on Saudi Arabia. Detailed explanations of the survey development, recruitment process, and non-response bias verification improve transparency and replicability. Additionally, expanded discussions on statistical methods and more descriptive statistics enhance the rigor and clarity of data analysis, making the manuscript more robust and accessible.

Are sufficient details of methods and materials provided to allow replication by others?

Partly

Is the rationale for creating the dataset(s) clearly described?

Yes

Are the datasets clearly presented in a useable and accessible format?

Yes

Are the protocols appropriate and is the work technically sound?

Partly

Reviewer Expertise:

Islamic banking and finance, credit risk, corporate governance, audit quality,  financial reporting, financial derivatives, financial inclusion, risk management, behavioural finance, sustainability

I confirm that I have read this submission and believe that I have an appropriate level of expertise to confirm that it is of an acceptable scientific standard.

# Reviewer report: Reviewer response for version 2

**DOI:** 10.5256/f1000research.172543.r336505

**Published:** 2024-11-06

**Authors:** Ines Kateb

**Affiliations:** 1 LIGUE ISCAE, University of Manouba (Ringgold ID: 108051), Manouba, Tunis, Tunisia; 2 Department of Accounting, Umm Al-Qura University, Mecca, Saudi Arabia

Dear Authors,

 Thank you for your responsiveness and the attention given to incorporating my comments into the revised version of your manuscript. I especially appreciate the detailed clarifications you've provided, which enrich the methodology and improve the transparency of your study.

**Item Selection and Validation**: I am pleased to see that you enhanced the methodological section, detailing the selection and validation process of the questionnaire items by drawing on existing literature and expert input. This precision strengthens the theoretical foundation of your study and clarifies the development of items used to measure self-efficacy. 

**Statistical Methods for Data Description: **Thank you for the additional clarity on the descriptive analysis employed. Including basic statistics such as mean, standard deviation, maximum, and minimum for each variable enhances understanding of your sample characteristics and methodology, complementing your descriptive approach and aligning with your study's objectives.

**Replication Process and Recruitment Methods**: The improved description of data collection and response verification procedures, including the use of two-tailed t-tests to assess non-response bias, adds scientific rigor to your study. This also makes the process more accessible for future research and comparative studies. 

**Language Clarity and Consistency:** I commend your efforts to improve the readability of the text, making your research more accessible to an international audience.

**Conclusion: **I am confident that the revisions made significantly enhance the clarity and scientific rigor of your study. These adjustments satisfactorily address the concerns raised in my initial report, and I believe that your article is now ready for indexing and the next stage in the editorial process.

 Thank you once again for your dedication to improving this article.

 Best regards

Are sufficient details of methods and materials provided to allow replication by others?

Partly

Is the rationale for creating the dataset(s) clearly described?

Yes

Are the datasets clearly presented in a useable and accessible format?

Yes

Are the protocols appropriate and is the work technically sound?

Partly

Reviewer Expertise:

Accounting, Auditing, IFRS, Governance, Financial reporting, CSR and Sustainability.

I confirm that I have read this submission and believe that I have an appropriate level of expertise to confirm that it is of an acceptable scientific standard.

# Reviewer report: Reviewer response for version 1

**DOI:** 10.5256/f1000research.160105.r317074

**Published:** 2024-09-16

**Authors:** Jaizah Othman

**Affiliations:** 1 Imam Abdulrahman Bin Faisal University, Dammam, Eastern Province, Saudi Arabia

This study addresses a timely and relevant topic by examining Saudi auditors’ self-efficacy and proficiency in conducting virtual audits during the COVID-19 pandemic. This research provides valuable insights into how auditors in Saudi Arabia have adapted to the unprecedented challenges of the pandemic and thus fit well into global discussions on the disruption of professional practice. The aim of this study is clearly outlined and focuses on developing auditors' self-efficacy and remote auditing capabilities during this time. The data collection process has been clearly described. An online survey was conducted to obtain responses from 193 auditors registered with the Saudi Organization for Chartered and Professional Accountants (SOCPA). The inclusion of demographic data enriches the analysis and provides a deeper understanding of accountants' profiles and how these characteristics may influence their responses. In addition, the study complied with the ethical guidelines and was approved by the local research ethics committee at Shaqra University, and appropriate consent procedures were followed. 

 However, there are some areas in which the study could be improved. An important improvement is to provide more details regarding the sampling method. It is unclear whether the 193 respondents were selected through random sampling or other methods. Clarifying this issue would improve the technical rigor and replicability of this study. The study also benefits from a broader contextualization of its findings. A brief discussion of the global impact of COVID-19 on audit practice before narrowing the focus to Saudi Arabia would help international readers better understand the relevance of the study in a broader context. In terms of replicability, although the methods are adequately described, additional details about the recruitment process and sample selection would help future researchers replicate the study. More transparency in these steps would increase the usefulness of this study for comparative research. 

 Finally, the dataset is presented in a clear and accessible format. The tables summarizing the demographic data and key constructs are well-organized, and the availability of the dataset in a public repository (Mendeley Data Repository) adds significant value by enabling further research and comparative analysis. To summarize, while the study addresses an important and timely topic, it could be improved by methodological clarifications and a broader global context for its findings. These improvements would enhance the study's contribution to the understanding of the evolving role of auditors during the COVID-19 pandemic.

Are sufficient details of methods and materials provided to allow replication by others?

Partly

Is the rationale for creating the dataset(s) clearly described?

Yes

Are the datasets clearly presented in a useable and accessible format?

Yes

Are the protocols appropriate and is the work technically sound?

Partly

Reviewer Expertise:

Islamic banking and finance, credit risk, corporate governance, audit quality,  financial reporting, financial derivatives, financial inclusion, risk management, behavioural finance, sustainability

I confirm that I have read this submission and believe that I have an appropriate level of expertise to confirm that it is of an acceptable scientific standard, however I have significant reservations, as outlined above.

# Response

**Published:** 2024-09-25

**Authors:** Mahfoudh Mgammal

**Affiliations:** Accounting, Amran university, Amran, Amran, Yemen

We are very grateful for the suggestions and concerns that we believe are insightful and will contribute to the quality of our data note. We are grateful for your appreciation of the potential value of our dataset. Thus, we are grateful to address your comments/suggestions as follows: 

**Comment 1:** *It is unclear whether the 193 respondents were selected through random sampling or other methods. Clarifying this issue would improve the technical rigor and replicability of this study.*

**Response: **Thank you for pointing out this interesting comment because it is important to clarify the methods used for collecting the data. To address this comment, we have clearly articulated what is our population and how we reached the final number of participants (second paragraph in the Methods) as follows:

 We identified all registered Saudi auditors as our population framework. SOCPA, the accounting and audit profession regulatory body in Saudi Arabia, provides a list of registered auditors and their information. As of April 2021, the list of registered auditors includes 25 audit firms/offices associated with 417 auditors, including audit partners and audit managers. Thus, all registered Saudi auditors were our population in this study, and they all had the chance to participate in this study. Specifically, the questionnaire targeted 417 registered auditors of 25 audit firms or offices with SOCPA as of April 2021. The online questionnaire was shared by email with all registered firms requesting each firm or office to distribute the questionnaire to all auditors. To increase the number of participants, we emailed audit firms and offices with two gentle reminders. Between April and August 2021, researchers gathered data from 193 auditors in Saudi Arabia, comprising 46% of the entire population. We assigned two researchers to review all responses for incompleteness and for inappropriateness of answers. This review concluded that 193 responses were usable for analysis. Furthermore, the researchers verified the potential non-response bias issue by comparing the responses received in April with those received in August. Using a two-tailed t-test, the researchers found no variation in the responses across the two groups, suggesting the absence of this issue.

**Comment 2:** *The study also benefits from a broader contextualization of its findings. A brief discussion of the global impact of COVID-19 on audit practice before narrowing the focus to Saudi Arabia would help international readers better understand the relevance of the study in a broader context.*

**Response: **We thank you for commenting on this, and we are aware of the importance of adding more clarifications on the impact of COVID-19 on audit profession. To address this concern, we have added more discussions on the effect of COVID-19 on the audit (first paragraph in the introduction) as follows:

 The coronavirus disease 2019 (COVID-19) is a health pandemic that disrupts the lives of humans and corporate businesses. Hay, Shires, and Dyk (2021) advocate that the COVID-19 crisis is more likely to affect the auditing profession. In this crisis, auditors were put in an unprecedented situation to deliver their audit activities, as they used to carry out most audit activities in the present, which became impossible during the pandemic due to social distancing and homestay requirements (Baatwah & Al-Ansi, 2022). Furthermore, the COVID-19 pandemic requires audit firms across the world to revise their assessment of material misstatement risks (Al-Qadasi, Baatwah, & Omer, 2023). According to the International Auditing and Assurance Standards Board (IAASB) and the Financial Reporting Council (FRC), the COVID-19 pandemic has introduced new risks of material misstatements that require auditors to redesign their audit approaches and execute extensive efforts to reduce these risks (IAASB, 2020; FRC, 2020). Audit practitioners have also acknowledged several difficulties in conducting audit activities during the COVID-19 pandemic (PwC, 2020; KPMG, 2020).

**Comment 3:** *Additional details about the recruitment process and sample selection would help future researchers replicate the study. More transparency in these steps would increase the usefulness of this study for comparative research.*

**Response: **We thank you for pointing out this interesting comment, because it is important to add more validation to the dataset. To address this concern, we have expended the discussion on how the researchers recruit the data and describe the sample selection process (second and fifth paragraphs in the methods) as follows:

 We identified all registered Saudi auditors as our population framework. SOCPA, the accounting and audit profession regulatory body in Saudi Arabia, provides a list of registered auditors and their information. As of April 2021, the list of registered auditors includes 25 audit firms/offices associated with 417 auditors, including audit partners and audit managers. Thus, all registered Saudi auditors were our population in this study, and they all had the chance to participate in this study. Specifically, the questionnaire targeted 417 registered auditors of 25 audit firms or offices with SOCPA as of April 2021. The online questionnaire was shared by email with all registered firms requesting each firm or office to distribute the questionnaire to all auditors. To increase the number of participants, we emailed audit firms and offices with two gentle reminders. Between April and August 2021, researchers gathered data from 193 auditors in Saudi Arabia, comprising 46% of the entire population. We assigned two researchers to review all responses for incompleteness and for inappropriateness of answers. This review concluded that 193 responses were usable for analysis. Furthermore, the researchers verified the potential non-response bias issue by comparing the responses received in April with those received in August. Using a two-tailed t-test, the researchers found no variation in the responses across the two groups, suggesting the absence of this issue.

 Before sharing the questionnaire with auditors, we took several steps to improve our instrument's readability, validity, and reliability. First, the questionnaire was shared with four audit professors, four audit partners, and two senior auditors to measure the reliability and readability of the content. Based on these comments and suggestions, we amended the questionnaire. Second, given that the first draft of the questionnaire was in English, in consultation with experts, we translated the questionnaire into Arabic, as this is the native language of most auditors in Saudi Arabia. Finally, we filled out all questionnaire items in a Google Form and created a Google Form link to be shared with auditors because social distance restrictions and the preferences of individuals communicating remotely were common at the time of developing this questionnaire. Furthermore, using a Google Form link is a common method for collecting data in survey-based studies as it is associated with less effort and fewer errors. Thus, all 25 registered audit firms in Saudi Arabia received an online questionnaire via email using contact information obtained from the SOCPA website.    

 Once again, we would like to thank you for your thoughtful and insightful comments and suggestions and hope that the current version of the manuscript meets your expectations.

# Reviewer report: Reviewer response for version 1

**DOI:** 10.5256/f1000research.160105.r317069

**Published:** 2024-09-13

**Authors:** Ines Kateb

**Affiliations:** 1 LIGUE ISCAE, University of Manouba (Ringgold ID: 108051), Manouba, Tunis, Tunisia; 2 Department of Accounting, Umm Al-Qura University, Mecca, Saudi Arabia

The article explores Saudi auditors' self-efficacy during the COVID-19 pandemic, specifically focusing on their proficiency with virtual audits. The dataset includes responses from 193 auditors on self-efficacy, its sources (mastery experience, vicarious experience, social persuasion, and physiological/emotional states), and virtual audit skills. The article provides valuable insights into how auditors adapted to pandemic-related challenges, offering a unique dataset for researchers, policymakers, and capital market authorities.

 The authors clearly justify the creation of the dataset by highlighting the unprecedented challenges auditors faced during COVID-19. The rationale is well-grounded, as the study addresses how auditors adapted to remote audits, a practice not commonly used before the pandemic. The study's focus on self-efficacy and virtual audit proficiency fills a gap in the literature.

 While the methods are generally appropriate, the article could improve by providing more detail on how the survey items were selected and validated. Additionally, more clarity on the statistical methods used for data analysis would strengthen the paper's technical soundness. The language inconsistencies also suggest that minor revisions could improve overall clarity.

 Recommendation: Provide more explicit details about the selection and validation of survey items. Clarify the methodology used for analyzing the dataset, including any statistical tests. Address minor language inconsistencies to improve readability.

 While the survey design is well-explained, further details are needed for full replication. Specifically, more information about the survey's development process, including how items were adapted from previous research and validated, would be beneficial. Additionally, details about how the data was processed post-collection are lacking.

 Recommendation: Expand on the survey development and validation process to enhance transparency. Include more specifics on data processing steps to facilitate replication.

 The dataset is clearly presented and accessible. It is stored in a public repository, and the article provides a detailed breakdown of the variables, making it easy for other researchers to use. The accompanying tables and descriptive statistics further enhance the dataset's usability.

 To make the article scientifically sound, the authors must address the lack of clarity in the survey's development and validation process, as well as improve the technical details surrounding the analysis methods. Minor language corrections would also enhance clarity.

Are sufficient details of methods and materials provided to allow replication by others?

Partly

Is the rationale for creating the dataset(s) clearly described?

Yes

Are the datasets clearly presented in a useable and accessible format?

Yes

Are the protocols appropriate and is the work technically sound?

Partly

Reviewer Expertise:

Accounting, Auditing, IFRS, Governance, Financial reporting, CSR and Sustainability.

I confirm that I have read this submission and believe that I have an appropriate level of expertise to confirm that it is of an acceptable scientific standard, however I have significant reservations, as outlined above.

# Response

**Published:** 2024-09-25

**Authors:** Mahfoudh Mgammal

**Affiliations:** Accounting, Amran university, Amran, Amran, Yemen

We are very grateful for the suggestions and concerns that we believe are insightful and will contribute to the quality of our data note. We are grateful for your appreciation of the potential value of our dataset. Thus, we are grateful to address your comments/suggestions as follows:

**Comment 1**

*While the methods are generally appropriate, the article could improve by providing more detail on how the survey items were selected and validated.*

**Response**

 We are grateful for your insightful comment. To address this comment, we have improved our discussion on the development of the items and the process of data collection. For example, we have highlighted how the items are developed by documenting the main theory and the related literature (third and fourth paragraphs in the methods) and elaborated more discussion on the process of data collection (second paragraph in the methods) as follows:

 We identified all registered Saudi auditors as our population framework. SOCPA, the accounting and audit profession regulatory body in Saudi Arabia, provides a list of registered auditors and their information. As of April 2021, the list of registered auditors includes 25 audit firms/offices associated with 417 auditors, including audit partners and audit managers. Thus, all registered Saudi auditors were our population in this study, and they all had the chance to participate in this study. Specifically, the questionnaire targeted 417 registered auditors of 25 audit firms or offices with SOCPA as of April 2021. The online questionnaire was shared by email with all registered firms requesting each firm or office to distribute the questionnaire to all auditors. To increase the number of participants, we emailed audit firms and offices with two gentle reminders. Between April and August 2021, researchers gathered data from 193 auditors in Saudi Arabia, comprising 46% of the entire population. We assigned two researchers to review all responses for incompleteness and for inappropriateness of answers. This review concluded that 193 responses were usable for analysis. Furthermore, the researchers verified the potential non-response bias issue by comparing the responses received in April with those received in August. Using a two-tailed t-test, the researchers found no variation in the responses across the two groups, suggesting the absence of this issue. 

 In designing the questionnaire, we extensively reviewed the related prior research. For example, we used the social cognitive theory developed by Bandura [4] and adapted items from education studies (e.g. Usher & Pajares, 2009; Peura et al., 2021; Zhang, Zhu, & Su, 2023) to develop items related to selfـــefficacy and its traditional sources because studies on selfـــefficacy in the context of auditing are limited (Mohd Sanusi, Iskandar, Monroe, & Saleh, 2018). Furthermore, we referred to Teeter et al.’s (2010) analytical framework to create items on virtual audit proficiency. Given that the questionnaire items are used in prior literature from other domains, the adaption of these items involves wording modifications to fit the auditing context. However, the adaptation of these items was shared with professors and experienced audit partners.

 Drawing on existing literature, we identified eight key items to explore auditor selfـــefficacy during COVID-19. These items are measured using eight statements on a five-point Likert scale ranging from "strongly disagree" to "strongly agree." Similarly, we adapted existing measures for the four factors that influence self-efficacy: mastery experiences (3 items), verbal and social persuasion (4 items), vicarious experiences (3 items), and physiological and emotional states (3 items). These items also used a five-point Likert scale, ranging from "strongly disagree" to "strongly agree." In addition, we developed six new statements to assess how well auditors can use advanced technologies in their work (virtual audit proficiency). These items were rated on a five-point Likert scale ranging from "rarely" to "always." The specific wording of all the items is provided in Tables 2 and 3. As in any survey study, we included a section providing general information or demographic attributes of the respondents. These include the audit firm's position, age, education, experience, type of affiliated audit firm, and client type. Table 1 presents the respondents’ demographic attributes.

**Comment 2**

*Additionally, more clarity on the statistical methods used for data analysis would strengthen the paper's technical soundness.*

**Response**

 Many thanks for suggesting this to us. With due respect, we would like to note that this study reports more discerptions on the data by focusing on methods of collecting the data and the measurements of key variables. We have used basic statistical methods such as mean, standards deviation, max, and min statistics. Thus, given this nature, we do not use more advance statistics that are common in testing hypothesis. Nonetheless, we have now clearly articulate these statistics in addition to any relevant statistics for descripting the data (second paragraph in the Data Description and second paragraph in the Methods)

 Table 4 reports the descriptive statistics for each construct using basic statistics such as the mean, standard deviation, maximum, and minimum.  

 Using a two-tailed t-test, the researchers found no variation in the responses across the two groups, suggesting the absence of this issue.

**Comment 3**

*The language inconsistencies also suggest that minor revisions could improve overall clarity.*

**Response**

 We really appreciate this insightful comment. To address this comment, we have proofread the paper and improve the presentation of the paper. We believe that the paper is now much improved.

**Comment 4**

*While the survey design is well-explained, further details are needed for full replication. Specifically, more information about the survey's development process, including how items were adapted from previous research and validated, would be beneficial. Additionally, details about how the data was processed post-collection are lacking.*

**Response**

 Thank you for pointing out this interesting comment because it is important to clarify the methods for replication this dataset. With due respect, we would like to note that we have improved our discussion on the development of the items and the process of data collection. As you can see in our response for the comment 1, we reported how this improvement is incorporated in the paper (please refer to our response for comment 1).

**Comment 5**

*To make the article scientifically sound, the authors must address the lack of clarity in the survey's development and validation process, as well as improve the technical details surrounding the analysis methods. Minor language corrections would also enhance clarity.*

**Response**

 Thank you for pointing this out interesting comment to us. With due respect, we would like to note that this comment summarized all comments/suggestions that raised before. In previous comments, we have tried our best to address these concerns and incorporated all these amendments in the paper. Thus, we will be grateful if you can refer to these previous comments and our responses.

 Once again, we would like to thank you for your thoughtful and insightful comments and suggestions and hope that the current version of the manuscript meets your expectations.

# Reviewer report: Reviewer response for version 1

**DOI:** 10.5256/f1000research.160105.r317075

**Published:** 2024-09-10

**Authors:** Che Siti Lazrina Md Lazim

**Affiliations:** 1 Commerce and Accountancy, Universiti Tunku Abdul Rahman, Kampar, Perak, Malaysia

This article provides a detailed overview of the study on external auditors in Saudi Arabia during the COVID-19 pandemic. The study addresses a critical issue—how the COVID-19 pandemic impacted auditing practices and is highly relevant to the ongoing disruptions caused by the pandemic. The introduction clearly states the purpose of the study, which is to understand auditors' self-efficacy and proficiency with virtual audits during the pandemic. So, the introduction is well articulated. Then, the methods section demonstrates a thoughtful approach to designing the survey, including reviewing existing literature and adapting validated measures. The steps taken to ensure the validity and reliability of the questionnaire, such as consultations with experts and translation into Arabic, are commendable. The data description section is thorough, detailing the structure of the dataset and the types of information collected. The inclusion of demographic data and various constructs related to self-efficacy and virtual audit proficiency adds depth to the analysis.

 However, the phrase “there has been not much studies addicted to” should be revised to “there have not been many studies addressing.” Another issue to be suggested is to provide a brief overview of the impact of COVID-19 on auditing practices globally before focusing on Saudi Arabia. This would offer readers a broader context. While, the methods section could benefit from a clearer explanation of the survey's development process, including how the items were selected and validated to enhance transparency.

 There are some minor language inconsistencies and awkward phrasing that could be improved for clarity. For instance, “The Local Committee of Research Ethics at Shaqra University (HAPO- 01-R-128)” could be more seamlessly integrated into the text. Overall, this article is well-structured and provides valuable insights into the challenges faced by auditors during the COVID-19 pandemic. By addressing the minor issues related to clarity, detailed presentation of results, and ensuring all tables are properly included, the article would be even more effective in communicating its findings.

Are sufficient details of methods and materials provided to allow replication by others?

Partly

Is the rationale for creating the dataset(s) clearly described?

Yes

Are the datasets clearly presented in a useable and accessible format?

Yes

Are the protocols appropriate and is the work technically sound?

Partly

Reviewer Expertise:

Accounting and Education Technology , Small Medium Enterprises

I confirm that I have read this submission and believe that I have an appropriate level of expertise to confirm that it is of an acceptable scientific standard, however I have significant reservations, as outlined above.

# Response

**Published:** 2024-09-25

**Authors:** Mahfoudh Mgammal

**Affiliations:** Accounting, Amran university, Amran, Amran, Yemen

We are very grateful for the suggestions and concerns that we believe are insightful and will contribute to the quality of our data note. We are grateful for your appreciation of the potential value of our dataset. Thus, we are grateful to address your comments/suggestions as follows: 

**Comment 1**

*However, the phrase “there has been not much studies addicted to” should be revised to “there have not been many studies addressing”.*

**Response**

 We are grateful for your insightful comment. To address this comment, we have revised this phrase as suggested (second paragraph in the introduction) as follows:

 Although the COVID-19 crisis raised concerns about the quality of audits and increased the risk of material misstatement, there have not been many studies addressing how accounting scholars have collectively contributed to our understanding of the challenges created by the COVID-19 (Mgammal, Al-Matari, & Bardai, 2022; Rinaldi, 2022).   

**Comment 2**

*Another issue to be suggested is to provide a brief overview of the impact of COVID-19 on auditing practices globally before focusing on Saudi Arabia. This would offer readers a broader context.*

**Response**

 Thank you for pointing this out. As suggested, we have highlighted the impact of COVID-19 on auditing practices by sharing the concerns of auditing regulatory authorities, audit practitioners, and audit scholars (first paragraph in the introduction) as follows:

 The coronavirus disease 2019 (COVID-19) is a health pandemic that disrupts the lives of humans and corporate businesses. Hay, Shires, and Dyk (2021) advocate that the COVID-19 crisis is more likely to affect the auditing profession. In this crisis, auditors were put in an unprecedented situation to deliver their audit activities, as they used to carry out most audit activities in the present, which became impossible during the pandemic due to social distancing and homestay requirements (Baatwah & Al-Ansi, 2022). Furthermore, the COVID-19 pandemic requires audit firms across the world to revise their assessment of material misstatement risks (Al-Qadasi, Baatwah, & Omer, 2023). According to the International Auditing and Assurance Standards Board (IAASB) and the Financial Reporting Council (FRC), the COVID-19 pandemic has introduced new risks of material misstatements that require auditors to redesign their audit approaches and execute extensive efforts to reduce these risks (IAASB, 2020; FRC, 2020). Audit practitioners have also acknowledged several difficulties in conducting audit activities during the COVID-19 pandemic (PwC, 2020; KPMG, 2020).  

 We have also added discussions highlighting more information on the Saudi setting that will enrich the suitability of using this setting (second paragraph in the introduction) as follows:

 The accounting and audit profession in Saudi Arabia has recently witnessed considerable development in regulatory frameworks and practices. For example, international accounting standards and international audit standards are references for firms and auditors to practice auditing and accounting activities. It also formulated an independent regulatory body, SCOPA, to develop and monitor auditing accounting practices in Saudi Arabia. However, similar to auditors in other countries, Saudi auditors experienced anomalies in conducting audit activities during the pandemic. Thus, several groups, such as regulators, those using financial data, policymakers, and researchers may be more interested in this dataset.

**Comment 3**

*While, the methods section could benefit from a clearer explanation of the survey's development process, including how the items were selected and validated to enhance transparency.*

**Response**

 We appreciate this insightful comment because we believe it adds value to our research. We have clearly indicated discretions on how the items of the instruments are developed (third and fourth paragraphs in the methods) as follows:

 In designing the questionnaire, we extensively reviewed the related prior research. For example, we used the social cognitive theory developed by Bandura [4] and adapted items from education studies (e.g. Usher & Pajares, 2009; Peura et al., 2021; Zhang, Zhu, & Su, 2023) to develop items related to selfـــefficacy and its traditional sources because studies on selfـــefficacy in the context of auditing are limited (Mohd Sanusi, Iskandar, Monroe, & Saleh, 2018). Furthermore, we referred to Teeter et al.’s (2010) analytical framework to create items on virtual audit proficiency. Given that the questionnaire items are used in prior literature from other domains, the adaption of these items involves wording modifications to fit the auditing context. However, the adaptation of these items was shared with professors and experienced audit partners.

 Drawing on existing literature, we identified eight key items to explore auditor selfـــefficacy during COVID-19. These items are measured using eight statements on a five-point Likert scale ranging from "strongly disagree" to "strongly agree." Similarly, we adapted existing measures for the four factors that influence self-efficacy: mastery experiences (3 items), verbal and social persuasion (4 items), vicarious experiences (3 items), and physiological and emotional states (3 items). These items also used a five-point Likert scale, ranging from "strongly disagree" to "strongly agree." In addition, we developed six new statements to assess how well auditors can use advanced technologies in their work (virtual audit proficiency). These items were rated on a five-point Likert scale ranging from "rarely" to "always." The specific wording of all the items is provided in Tables 2 and 3. As in any survey study, we included a section providing general information or demographic attributes of the respondents. These include the audit firm's position, age, education, experience, type of affiliated audit firm, and client type. Table 1 presents the respondents’ demographic attributes.

 We have also added more elaboration on the process of collecting the data (second paragraph in the methods) as follows:

 We identified all registered Saudi auditors as our population framework. SOCPA, the accounting and audit profession regulatory body in Saudi Arabia, provides a list of registered auditors and their information. As of April 2021, the list of registered auditors includes 25 audit firms/offices associated with 417 auditors, including audit partners and audit managers. Thus, all registered Saudi auditors were our population in this study, and they all had the chance to participate in this study. Specifically, the questionnaire targeted 417 registered auditors of 25 audit firms or offices with SOCPA as of April 2021. The online questionnaire was shared by email with all registered firms requesting each firm or office to distribute the questionnaire to all auditors. To increase the number of participants, we emailed audit firms and offices with two gentle reminders. Between April and August 2021, researchers gathered data from 193 auditors in Saudi Arabia, comprising 46% of the entire population. We assigned two researchers to review all responses for incompleteness and for inappropriateness of answers. This review concluded that 193 responses were usable for analysis. Furthermore, the researchers verified the potential non-response bias issue by comparing the responses received in April with those received in August. Using a two-tailed t-test, the researchers found no variation in the responses across the two groups, suggesting the absence of this issue.  

**Comment 4**

*There are some minor language inconsistencies and awkward phrasing that could be improved for clarity. For instance, “The Local Committee of Research Ethics at Shaqra University (HAPO- 01-R-128)” could be more seamlessly integrated into the text.*

**Response**

 Thank you for highlighting this concern. To address this comment, we have moved this statement to the end parts of this paper under section “Ethics and Consent Statement”. Furthermore, we have proofread the paper to improve the language and presentation (please check throughout the paper).

 Once again, we would like to thank you for your thoughtful and insightful comments and suggestions and hope that the current version of the manuscript meets your expectations.
